# A Hybrid Process for Printing Pure and High Conductivity Nanocrystalline Copper and Nickel on Flexible Polymeric Substrates

**DOI:** 10.1038/s41598-019-55640-7

**Published:** 2019-12-13

**Authors:** Md Emran Hossain Bhuiyan, Ali Behroozfar, Soheil Daryadel, Salvador Moreno, Seyedreza Morsali, Majid Minary-Jolandan

**Affiliations:** Department of Mechanical Engineering, The University of Texas at Dallas, Richardson, TX USA

**Keywords:** Engineering, Materials science

## Abstract

Printing functional devices on flexible substrates requires printing of high conductivity metallic patterns. To prevent deformation and damage of the polymeric substrate, the processing (printing) and post-processing (annealing) temperature of the metal patterns must be lower than the glass transition temperature of the substrate. Here, a hybrid process including deposition of a sacrificial blanket thin film, followed by room environment nozzle-based electrodeposition, and subsequent etching of the blanket film is demonstrated to print pure and nanocrystalline metallic (Ni and Cu) patterns on flexible substrates (PI and PET). Microscopy and spectroscopy showed that the printed metal is nanocrystalline, solid with no porosity and with low impurities. Electrical resistivity close to the bulk (~2-time) was obtained without any thermal annealing. Mechanical characterization confirmed excellent cyclic strength of the deposited metal, with limited degradation under high cyclic flexure. Several devices including radio frequency identification (RFID) tag, heater, strain gauge, and temperature sensor are demonstrated.

## Introduction

Additive printing processes are promising for low-cost and more versatile device fabrication on flexible substrates in comparison with the conventional fabrication processes such as photolithography and high vacuum processes. In particular, the capability to directly print high conductivity metallic patterns on flexible substrates without a need for high temperature annealing will accelerate the progress of flexible electronics field^1^. To prevent deformation and damage of the polymeric substrate, the processing (printing) and post-processing (annealing) temperature of the metal patterns must be lower than the glass transition temperature of the substrate. Often, most plastic and paper substrates require processing temperature below 150 °C^2^, since high processing temperature degrades their mechanical integrity.

To date, there are several microscale metal printing processes such as direct ink writing (DIW)^3–5^, electrohydrodynamic printing (EHD)^6,7^, laser-induced forward transfer (LIFT)^8,9^, focused electron/ion beam induced deposition (FEBID/ FIBID)^10^, and laser-induced photoreduction (LIPR)^11,12^. Among these processes, to our knowledge, so far DIW and EHD processes have been investigated for printing metals directly on flexible substrates^4,13^. DIW and EHD often require sintering to remove the solvent and binders. Removal of the solvent and binders may result in porous structures, and compromise the electrical and mechanical properties of the printed metal.

Recently, significant progress has been made for room-temeprature annealing of printed inks. Low pressure argon plasma has been used to sinter inkjet printed silver tracks on polymeric substrates^14^. The substrate temperature right after plasma exposure was measured to be ~71 °C. This method resulted in resistivity less than 10-time higher than the bulk value of silver. Conductive inks with a “built-in” sintering mechanism, which is triggered during drying of the printed pattern have been demonstrated. These inks achieve over 40% of the conductivity of bulk silver^15^. Photonic sintering of silver nanoparticles ink has been also demonstrated, which achieves fast (~15 s) sintering rate and high electrical conductivity (40% of bulk silver)^16^. Another method is spontaneous coalescence of silver nanoparticles when they come into contact with oppositely charged polyelectrolytes. This method achieves 20% of the bulk silver conductivity^17^. Particle-free, reactive silver inks has been reported that achieve electrical conductivity equivalent to that of the bulk silver after sintering at 90 °C^18^. Apart from DIW and EHD process, other available microscale metal printing processes such as LIFT and LIPR use a laser, which may not be compatible with flexible substrates. Organic material contamination happens in the FEBID/FIBID process, and the high carbon content causes inferior mechanical and electrical properties of the as-deposited structures^19^. This process also requires thermal annealing at higher temperatures for several hours^20^.

**Figure 1:**
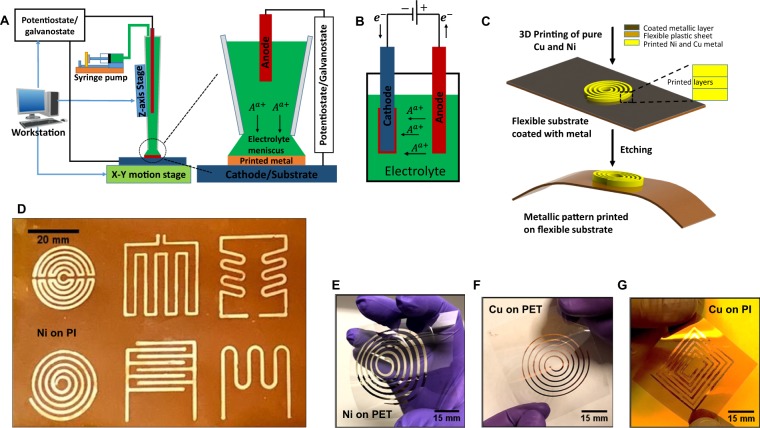
(**A**) The schematic illustration of the process. The close-up view of the electrolyte meniscus between the printing nozzle and the substrate. (**B**) The schematic of the conventional bulk electroplating process is shown for comparison. (**C**) The schematic shows the hybrid process for printing pure metals on a flexible substrate. (**D**–**G**) Photographs of various metal (Ni and Cu) patterns printed on flexible substrates (PI and PET).

Here, a hybrid process is demonstrated to print high conductivity nanocrystalline pure metals (Nickel (Ni) and Copper (Cu)) on flexible substrates (polyimide (PI) and Polyethylene terephthalate (PET)), Fig. 1A–C. The process includes deposition of a sacrificial blanket thin film, followed by room environment nozzle-based electrodeposition, and subsequent etching of the blanket film. Microscopy and spectroscopy showed that the printed metal is nanocrystalline, solid with no porosity and with low impurities. Electrical resistivity close to the bulk (~2-time) was obtained without any thermal annealing. Mechanical characterization confirmed excellent cyclic strength of the deposited metal, with limited degradation under high cyclic flexure. The advantage of this method compared to microfabrication is that the printing process does not require different masks for different designs, and patterns can be easily controlled by a computer program. Compared to DIW and EHD, this process results in pure metal with no binders or solvents, and hence, does not require sintering or annealing. Additionally, using this process, we demonstrate potential applications for direct printing of radio frequency identification (RFID) tag, heater, strain gauge, and temperature sensor on flexible substrates (Fig. 1D–G).

The printer setup and the close-up view of the electrolyte meniscus between the nozzle and printed metal is schematically shown in Fig. 1A. The nozzle-based electrodeposition process is based on the conventional electroplating process, which is schematically shown in Fig. 1B for comparison. In comparison with the conventional electroplating process, a nozzle with a few microns to millimeters tip diameter, containing the electrolyte, functions as the printing head. This process was first introduced in 2010 for deposition of wire-bonds^21^. In this process, a wire electrode inserted into the nozzle works as the anode, and the conductive substrate functions as the cathode. The nozzle is placed close to the substrate using a set of precision positioning stages. The electrolyte meniscus is established when the nozzle approaches to the substrate and an ionic-electronic circuit is completed. The electrolyte bath forms a small liquid bridge between the nozzle tip and the cathode surface (substrate)^22,23^. The nozzle is moved on the substrate surface and pulls the liquid bridge on the surface. Once printing starts, metal ions are consumed and fresh electrolyte is fed into the nozzle and the liquid bridge by a syringe pump. The precise and controlled movement of the syringe tip and/or the stage results in printing of desired patterns. The patterns can be formed in one layer, and as the process is repeated, multiple layers can be printed. The thickness of the pattern can be controlled by the number of the printed layers and process parameters (e.g. current density and electrolyte concentration).

A sacrificial conductive layer was first evaporated on the polymer substrate. This is because electroplating requires a conductive surface. The undesired conductive layer from the substrate was removed using a wet etching process after the printing process (Fig. 1C). Commercially available metal cladded flexible substrates can be also used. Another method would be laser-induced selective activation of the polymer substrate for electroplating. A previous research has also demonstrated continuous printing from a conductive surface to a nonconductive surface, in which the conductive surface functions as the cathode^24^.

In electroplating, the deposition rate of a particular metal electroplating is controlled by current density, as well as by current efficiency. In this work, for the pattern fabrication, the current density was maintained at 35 mA/cm^2^ for the Cu printing and 25 mA/cm^2^ for the Ni printing. In this method, for a given current density and printing speed, the volumetric deposition remains relatively constant. The volumetric deposition is determined by the nozzle cross-sectional area and the thickness of the printed pattern. Hence, for the given nozzle diameter and printing speed, under a constant current, the thickness of the printed pattern will be constant, which is obtained from the Faraday’s law. To increase the thickness, we print multiple layers to desired thickness. We can also increase or decrease the thickness of single layer by changing the applied current density and printing speed.

Figure 1D–G show photographs of various printed metal (Ni and Cu) patterns. Two different substrates with different melting temperatures, of approximately 400 °C for PI and 150 °C for PET, were considered. Both of these substrates are common for flexible electronics applications. PET is of particular interest for flexible electronics and photovoltaic applications given its high transparency and chemical stability^25^. The patterns were printed at room temperature, and have three layers, and were printed with a rate of 0.1 mm/s. Printing in different scales and line thicknesses can be achieved by varying the nozzle size. As an example, microscale patterns on PI sheet is shown in Fig. S1. The printed lines are continuous and fairly uniform in width, which is important for fabrication of functional devices.

**Figure 2:**
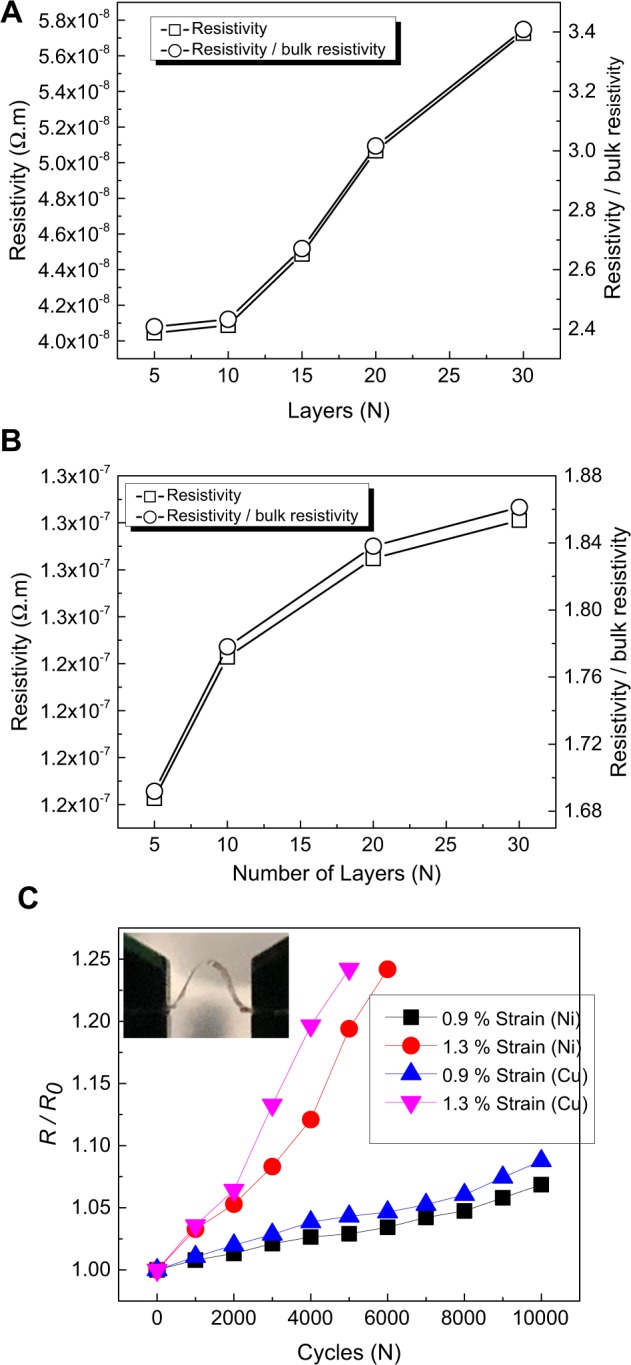
(**A**,**B**) The electrical resistivity and the normalized electrical resistivity (by the bulk resistivity) versus the number of the printed layers, for Cu and Ni, respectively. (**C**) The normalized resistance R/R_0_, where R_0_ designates the as-printed resistance before the flexural cycling, vs. the number of flexure cycles under two different flexural strains for Cu and Ni. The inset shows the side-view of the flexed substrate under 0.9% strain.

The electrical resistivity of the printed Cu and Ni was measured and compared with their bulk resistivity (Fig. 2A,B). Note that the conductivity of the underlying sputtered thin metal layer was subtracted, and the reported conductivity is for the printed metal only. The 5-layer Cu and Ni exhibited resistivity of ~4.0 × 10^−8^ Ω.m, and ~1.2 × 10^−7^ Ω.m, respectively, 2.4-time of the bulk resistivity (1.68 × 10^−8^ Ω.m) of Cu and 1.7-time of the bulk resistivity (6.99 × 10^−8^ Ω.m) of Ni. Such low electrical resistivity is impressive, given that no thermal annealing or sintering was performed on the printed metals. The effect of the printed layers number on the electrical resistivity was also investigated, as shown in Fig. 2A for Cu and 2B for Ni. The results show that resistivity increases slightly vs. the number of layers. For 30-layer, the electrical resistivity of Cu and Ni was ~3.4-time and ~1.9-time of the bulk resistivity, respectively. The increasing trend of the resistivity can be explained by considering a few factors. One possibility is oxidation of each layer before the next layer is printed. This is supported by the fact that the printed Ni shows much less increase in resistivity vs. the number of layers compared to Cu, which could be due to its much slower oxidation compared to Cu. Another possibility is microstructural defects in each printed layer that accumulate as the number of layers increases.

Detailed comparison of resolution and speed for various sub-micron metal printing processes are provided in a recent review article^26^. Overall, the chemical reaction-based methods offer higher resolution while slower speed compared to physical extrusion or jetting-based methods. During printing in a confined area, electrodeposition can be slower than bulk (bath) electrodeposition due to several factors including smaller cathodic surface area, and the absence of electrolyte agitation. In the LED process, several factors can affect the printing process, including optimizing the electrolyte bath parameters such as the electrolyte pH and concentration, as well as the applied current density^27^. Increasing the cathode surface temperature or electrolyte temperature, by applying external heat source, may also improve the speed of the process. In addition, deposition rate may increase by applying an external magnetic field^28^.

We also characterized changes in electrical resistance of the printed metal patterns on polymer substrate under cyclic loading. The cyclic test was performed by dynamic flexure experiment. Details explanation of experimental setup and strain calculation for the dynamic flexural test are provided in the supporting document (Figs. S2 and S3). Typical responses showing the evolution of the relative electrical resistance *R/R*_0_ (where *R*_0_ is the initial resistance of the printed line and *R* is the measured resistance during straining) of the printed metal as a function of cycle number are shown in Fig. 2C. The resistance increased by approximately 5% for 10,000 cycles under 0.9% peak flexural strain. Under 1.3% peak flexural strain, the resistance change increased to ~25% for 5,000 and 6,000 cycles for Cu and Ni, respectively. Considering that the initial resistivity of the printed patterns are very close to the bulk value, this change in resistance after large number of cycles is acceptable.

In addition to the cyclic test, conventional tape peel test was also performed on the printed Cu and Ni lines on the PI substrate to qualitatively assess the adhesion between printed metals and PI/PET substrates. Detail explanation of tape peeling test is included in the supplementary information and optical images of before and after tape peel test are shown in Fig. S5.

The surface morphology of the printed metals at different conditions (before and after flexure test) for the two different peak flexural strains are included in the supporting document (Fig. S4). The resistance increase during cyclic flexure loading is often described as the result of the mechanical damage accumulation. Typical fatigue-induced damage in the form of cracks was observed in SEM images, however, no clear through-thickness cracks were visible. These cracks are responsible for the increase of electrical resistance. In addition to the cracks, changes in the grain size and grain boundary contribution to the resistance could also be one of the reasons for the change in electrical resistance^29,30^.

Strain in a beam under bending is proportional to the thickness of the beam (*ε* = *y/p*), in which *y* is the thickness and *ρ* is the radius of curvature. The small value of strain is only because the thickness of the PI sheet and the printed metal is small. Additionally, although, the applied strain is small, the failure mechanism is not similar to the ductile metals. Research on ductility of thin metal films on polymer substrates has found that the metal film can rupture at strains ranging from a few percent to a few tens of percent. This variation in the ductility of the metal film is modulated by the adhesion of the metal/polymer interface^31^.

The failure of metal thin films on polymeric substrates can be explained considering possible failure mechanisms. Often, with the application of strain, the cracks form by a mixture of strain localization and intergranular fracture^32^. If the adhesion between the metal film and polymeric substrate is good, the metal film deforms uniformly, without traction in between the film/substrate interface. This uniform deformation suppresses the strain localization in the film with large percent of strain. Weak adhesion between the metal film and the substrate facilitates debonding and failure at lower strains, without suppressing strain localization. Imperfections in the film also initiate strain localization, traction in between film/substrate interface, and delaminate the metal film. The two processes, strain localization and delamination, promote each other and coevolve, leading to the propagation of the channel cracks. Mismatch of elastic modulus between metal film and substrate also resist to suppress the strain localization, if the substrate is too complaint with respect to metal film^33^.

**Figure 3:**
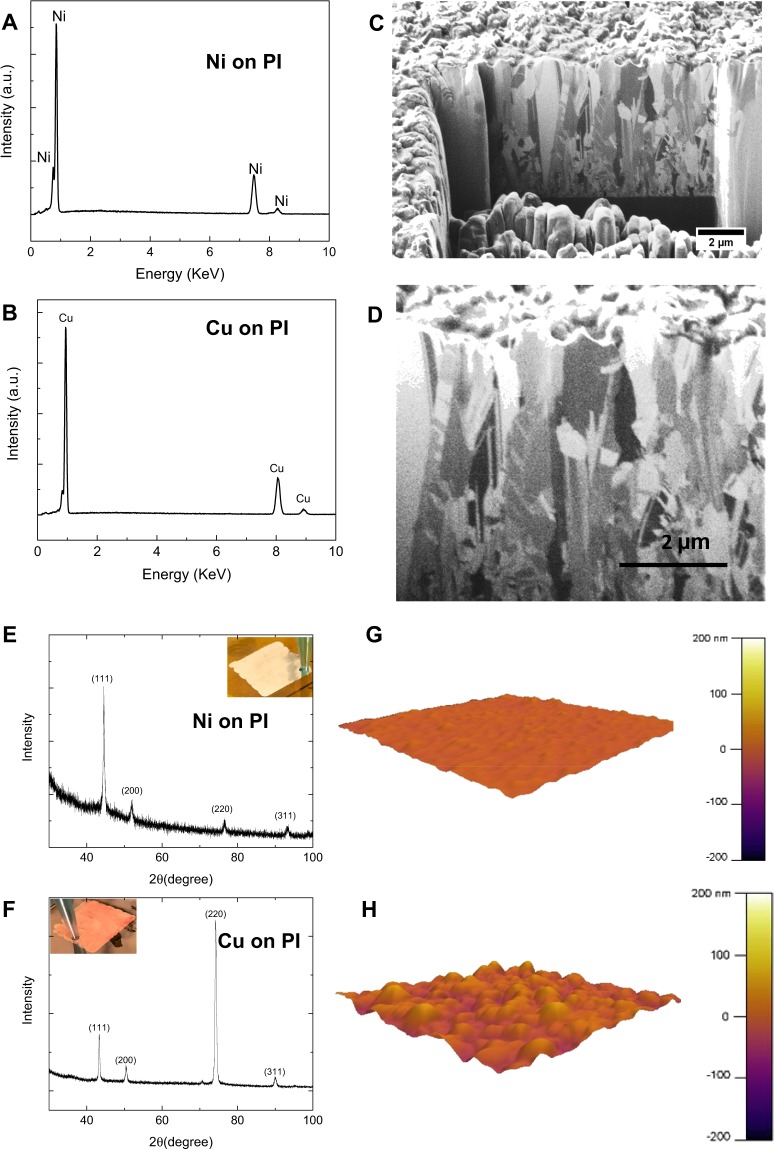
(**A**,**B**) The EDS spectra of the printed Ni and Cu on the PI sheet, respectively. (**C**,**D**) Cross-section FIB images of the printed Cu and Ni on the PI sheet. XRD spectra of (**E**) Ni and (**F**) Cu printed on the PI substrate. (**G**,**H**) AFM topography images of the printed Ni and Cu on the PI sheet, respectively.

Elemental composition was assessed by the EDS (Energy dispersive X-ray Spectroscopy). The EDS spectra are shown in Fig. 3A,B for Ni and Cu on the PI substrate, and in Fig. S6 on the PET substrate. The EDS spectra revealed that both metals (Cu and Ni) on both substrates do not have any detectable impurities such as sulfur (which may be incorporated into the deposited metal from the electrolyte). The EDS spectra of Cu shows a small shoulder next to the large peak ~1 keV energy, which is attributed to a small amount of oxygen, since Cu tends to rapidly oxidize in air^34,35^.

Electrical and mechanical properties of metals are often affected by the presence of pores and defects. The cross-section of the printed metal was cut by the focused ion beam (FIB) milling, and ion channeling contrast images were acquired (Fig. 3C,D). Cross-section FIB images show that printed metals are fully dense with low or no porosity. Additionally, FIB images reveal that majority of the grains are columnar, with the grains elongated along the electric field direction during the deposition process. More interestingly, the FIB cross-section images revealed that there were no noticeable interlayer between the printed layers, as if the metal was grown epitaxially rather than layer-by-layer. The absence of noticeable interlayer may be another reason for low electrical resistivity of the printed metals.

XRD (X-ray diffraction) spectra were acquired from the printed metals (Fig. 3E,F). The spectra show both Cu and Ni are crystalline. The spectra contains (111), (200), (220), and (311) peaks, indication of FCC (face centered cubic) crystal structure. Ni has a strong (111) peak. For Cu the strongest peak is (220), followed by (111) peak. In FCC metals, the {111} plane is the closed pack plane, and has the highest electromigration resistance. Therefore, the printed Cu and Ni can be used in high current density applications. Based on XRD analysis and using the Scherer′s equation, the estimated crystallite sizes of Ni and Cu with preferred orientation (111) and (220) were found to be 20 nm and 21 nm, respectively. Surface roughness analysis obtained from AFM images also confirmed the nanocrystalline nature of the printed Ni and Cu lines. The root mean square (RMS) values were found to be approximately 5 nm and 18 nm for the printed Ni and Cu (Fig. 3G,H, Figs. S7 and S8). Based on the FIB cross-section images (Fig. 3C,D), the grains are columnar in transverse direction. Hence, overall the printed metal has columnar nanocrystalline texture. By changing the process parameters, it should be possible to control the microstructure of the printed metal to a great extent^27,34,35^.

The thickness of the printed metal vs. the number of layers is shown in Fig. S8A,B. Overall, the printed Cu has a larger thickness for the same number of layers (>3-times) compared to Ni. This can be attributed to the higher growth rate for Cu compared to Ni. Additionally, based on AFM surface topography images (Fig. 3G,H) and Fig. S8, Cu has nearly 5-time larger surface roughness compared to Ni. Additionally, for both metals the RMS surface roughness increases with the number of layers. The initial roughness of the flexible polymeric substrates and roughness of the previously printed layer may contribute to the roughness of the subsequent layer. In addition, the alignment of the crystal planes in the printed material may also contribute to the observed surface roughness. We also examined the effect of the conductive layer etching on the printed metal surface roughness. RMS data shows that etching process slightly increases the surface roughness of the printed metal. Overall, 30-layers Ni and Cu printed on PET and PI substrates show maximum RMS roughness values lower than ~60 nm and ~12 nm, respectively, which is lower than other microscale metal printing methods^26^.

As proof of concept, several applications of the printed metallic patterns on the flexible substrates were demonstrated. These devices included a radio frequency identification (RFID) tag, a strain gauge, a temperature sensor, and a heater, as discussed below.

### RFID tag

**Figure 4:**
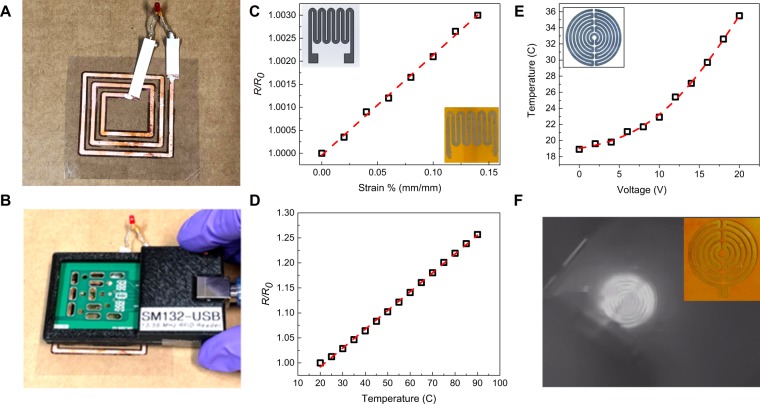
(**A**) An RFID tag made of printed Cu on PET sheet. (**B**) An LED is lit up using wireless power from the RFID reader. (**C**) Sensitivity response of a printed Ni strain gauge. (**D**) Response of the printed Ni temperature sensor. (**E**) Temperature change of the printed Ni heater vs. applied voltage. (**F**) An IR-camera image of the heated metal on the PI sheet. The inset shows the actual heater on the PI sheet.

RFID tags have applications in different sectors including medical and military for object tracking and scanning purposes^36^. We demonstrate an RFID tag by directly printing Cu on a PET substrate (Fig. 4A). An LED with a 100 pF capacitor was connected to the RFID tag to show its functionality. The RFID tag was powered wirelessly by a RFID reader, which transmits at 13.56 MHz. Figure 4B shows the LED (~1 mW) that was wirelessly powered by the RFID tag at a distance of ~15 mm from the reader. When the RFID reader is brought closer to the RFID tag, the brightness of the LED increased. The LED completely turned off at a distance of ~20 mm from the reader.

### Strain gauge

Strain gauges are widely used in measuring force, pressure, and displacement. A Ni strain gauge pattern based on commercial strain gauges was printed on a PI sheet (Fig. 4C, inset). The strain gauge was calibrated using a cantilevered beam (Fig. S9). The calibration results showed a linear relation between the strain and the resistance change up to ~0.15% strain. This limit was based on the experimental setup of the cantilever flexure frame, and not the PI sheet or the printed metal. The experimental gauge factor (GF) for this strain gauge was calculated to be ~2.14.

### Temperature sensor and heater

Flexible temperature sensors have potential applications in healthcare monitoring and personal electronics devices^37^. The change in resistance was used for the demonstration of a temperature sensor with the same design of the strain gauge. Figure 4D shows a linear relation between the temperature and the resistance from room temperature to 90 °C. The temperature sensor calibration procedure is discussed in detail in the materials and method section, and details of the experimental setup is shown in Fig. S10B. The slope of the linear response for the temperature sensor is ~0.0037 °C^−1^, and the sensitivity of the temperature sensor is ~0.20 Ω. °C^−1^. Since the resistor generates heat when current passes through it, the printed metal on flexible substrates could be used as a heater. A Ni heater pattern similar to the commercially available design was printed on a PI sheet as shown in the inset of Fig. 4E. A voltage in the range of 0–20 V was applied to the heater, which caused a temperature increase to 36 °C at 20 V. The infrared (IR) image (Fig. 4F) shows heat radiating from the metal element. The heater performance is fitted with a 2^nd^ order polynomial function. By changing the thickness of the metal pattern, and hence the resistivity of the printed lines, the sensitivity of the fabricated devices can be controlled based on the application requirements.

## Conclusions

In conclusion, the work presented here demonstrates a room environment process for additive printing of pure nanocrystalline metals on flexible substrates. The process requires a sacrificial conductive layer to be deposited on the polymer substrate and subsequently etched away after the printing process. The distinct advantage of this process is that it does not require thermal annealing or sintering process for the printed metal to become fully dense or electrically conductive. FIB, EDX, and XRD results showed that fully dense and pure metallic patterns can be printed. Without any thermal annealing, the printed Cu and Ni features exhibited nearly bulk electrical resistivity, which is remarkable. Applications of the process for fabrication of functional devices including RFID tags, strain gauges, heater and temperature sensors on flexible substrate were demonstrated.

## Experimental Section

### Materials

For Cu printing, the Cu electrolyte was composed of 1 M Copper (II) sulfate pentahydrate and 1 M of sulfuric acid in DI water. The anode was a copper wire with a diameter of approximately 0.3 mm inserted into the plastic syringe from the backside. For the Ni printing, the classical Watts’s bath was used^38^. The electrolyte was composed of 0.5 M of Nickel (II) sulfate hexahydrate (Sigma-Aldrich), 0.1 M of Nickel (II) chloride (Sigma-Aldrich) and 0.7 M of Boric acid (Sigma-Aldrich) in DI water. The anode was a Nickel foil (40 mm × 10 mm × 0.5 mm with a purity of %99.98, Sigma-Aldrich), which was inserted into the plastic syringe. The printing substrates (cathode) were 50 µm thick polyimide foil (Kapton 50HN by DuPont) and 50.8 µm thick PET foil. Cu etchant (APS-100 from TRANSENE) consisting of 15–20% Ammonium peroxydisulfate, and 80–85% water by weight was used for etching the conductive coating after printing. Ni etchant (TFG type from TRANSENE) consisting of less than 1% Thiourea, 10–15% Sodium n-nitrobenzene sulfonate, less than 10% sulfuric acid, and more than 75% water by weight was used. Both etchants were used without further diluting their concentration.

### The printer setup

The printing nozzles were 1.2 mm and 0.7 mm plastic pipettes, mounted on a 1 ml plastic syringe, which contained the electrolyte and the anode. This small syringe was connected to a larger syringe connected to a syringe pump (NE-300 New Era Pump). The nozzle was mounted on a z-stage, and the substrate was mounted on a xy-stage, and all stages were controlled using the same controller. G-code was used to control the nozzle path. A VersaSTAT-4 Potentiostat/Galvanostat (Princeton Applied Research) was used to apply the current/voltage.

### Pattern printing and etching

To prepare the substrate for electrodeposition, a 30 nm conductive layer of Cu and Ni was deposited by using an e-beam vapor deposition system at a base pressure better than 5 × 10^−6^ Torr with a deposition rate of 0.4 angstrom/sec. Each coating was used for the corresponding metal printing (Cu on Cu and Ni on Ni). Immediately prior to the deposition of Cu and Ni, the substrate was ultrasonically cleaned with acetone and isopropanol. For the pattern fabrication, the current density was maintained at 35 mA/cm^2^ for the Cu printing and 25 mA/cm^2^ for the Ni printing. The nozzle speed (printing speed) was set to 0.1 mm/s for both Cu and Ni printing. The mentioned parameters were achieved after a set of experiments and microscopic analysis on the printed metals and found to be optimum for the LED process in this scale. To remove the e-beam evaporator deposited Cu and Ni as the conductive layer, wet etching was performed using Cu and Ni etchant. For the Cu etching, it was performed at room temperature, and only 3–4 seconds was required to remove the 30 nm Cu layer. Ni etching was performed at 50 °C for approximately 90 seconds to remove the 30 nm Ni layer.

### Conductivity measurement

30 nm Cu for Cu-printing and Ni for Ni-printing were deposited on a substrate. Next, 10 mm linear Cu and Ni lines were printed with varying layer number. After printing, the entire substrate was etched to remove the initial deposited Cu and Ni from the unprinted part. Wet etching was used in this work. Then, the resistance of the 10 mm long wire was measured using an Agilent 33410a multimeter in the two-probe configuration. To calculate the electrical resistivity, the average cross-sectional area was measured by a profilometer. The resistance of the 30 nm deposited Cu/Ni layer under the printed line was subtracted from the total resistance.

To subtract the resistance of the initial e-beam deposited layer, we considered the printed line and the evaporated line as two different resistors connected in parallel, and the parallel resistance $(\frac{1}{{R}_{total}}=\frac{1}{{R}_{LEDprocess}}+\frac{1}{{R}_{e-beamprocess}})$ equation was used to obtain the resistance of the printed line by LED process. To calculate the electrical resistivity, the average cross-sectional area was measured by a profilometer, and the initial 30 nm e-beam deposited area was subtracted from the average cross-sectional area. Electrical resistivity was calculated using the following equation $(\rho =\frac{RA}{L})$. For example, for a 5-layer printed Cu line, the resistance of the e-beam deposited layer was ~12 Ω, and measured resistance of the printed line was ~0.12 Ω with the cross-sectional area $A=3.33\times {10}^{-3}\,m{m}^{2}$, after subtracting the cross-sectional area of initial 30 nm e-beam deposited Cu. For 30 layers Ni line, initial resistance of the e-beam deposited layer was 63.5 Ω, and measured resistance of the printed line was 0.46 Ω with cross-sectional area $=2.81\times {10}^{-3}\,m{m}^{2}$, after subtracting the cross-sectional area of the initial 30 nm e-beam deposited Ni.

### FIB sample preparation

10-layer Cu and Ni lines were printed on a PI sheet. After polishing and cutting the cross-section by using a dual beam FEI NOVA 200 FIB, ion channeling contrast images were acquired from the polished surface. The beam voltage was set at 30 kV and three different currents including high current (5 nA), medium current (1 nA), and low current (0.1 nA) were applied to cut the cross-section in several steps. Ion channel images were acquired using low energy ion beam (30 KV, 10 pA).

### Thickness measurement

Profilometer (Veeco Dektak VIII) was employed to measure the thickness of the printed Cu and Ni lines. Base line correction was made for all the measurements. At least three profilometer scans were taken for each thickness measurement.”

### XRD sample preparation and experiment

The crystal structure of the printed metals were studied by XRD. A 10 mm × 10 mm size (consisting of 10 overlaying lines in 5 layers) sample was printed for both Cu and Ni. Ni and Cu were printed on the PI substrate. To make the sample surface flat, the PI substrate was firmly attached to a small glass slide. XRD measurements were performed in a Rigaku smartlab XRD using a Cu Kα radiation with a wavelength of *λ* = 0.15406 nm in the range of 2*θ* = 30°–100°. Scanning step size and speed were *Δ*(2*θ*) = 0.01° and 1 deg/min, respectively. The voltage and current were set at 40 kV and 44 mA, respectively.

### AFM and EDX sample preparation and experiment

The surface roughness analysis was performed using an Asylum Bio3D Atomic Force Microscopy (AFM). Cu and Ni lines were printed on the PI and PET substrates. The PI and PET substrates were firmly attached to a small glass slide for better scanning. To obtain the RMS value of the surface roughness, at least three scans were performed for each sample at different locations. AFM images were recorded over scan area of 5 µm × 5 µm with a resolution of 512 × 512 pixels. The elemental analysis was conducted using a ZEISS Supra 40 scanning electron microscope (SEM). The EDS analysis was done at least three times at different points for each sample.

### Strain sensor fabrication and calibration

The printed strain sensor made of Ni was calibrated using a cantilevered beam under controlled beam deflection. To firmly attach the strain sensor to the Aluminum cantilever, a very thin epoxy glue layer was used, in addition to a single-sided tape at the top to assure profile uniformity. Copper tape soldering terminals were attached to connect thin wires to the two ends of the sensor. The beam was deflected using a cantilever flexure frame (Vishay) while measuring the deflection with a micrometer (Starrett). The resistance change with respect to the beam deflection was measured using an Agilent 33410a multimeter in two-probe configuration. Strain versus resistance was calibrated using beam deflection measurements and the elastic beam theory.

### Temperature sensor fabrication and calibration

The Ni electrode was printed on a PI substrate with the similar design to the strain gauge. For sensitivity calibration, it was attached onto an aluminum heat sink block, connected to a controller, by double-sided copper tape to maintain good thermal contact and uniform contact of the substrate with the block. The temperature of the aluminum block was increased from 20 °C to 90 °C while recording the temperature from the digital display of the controller. The temperature-dependent resistance of the metal electrode was recorded using an Agilent 33410a multimeter in the two-probe configuration.

### Heater pattern fabrication and characterization

A heater pattern with similar design of a commercially available heater was printed onto a polyimide sheet using the Ni electrolyte. Copper tapes with thin wires were soldered onto the two terminals of the heater pattern to connect the heater pattern to the power supply. A Keithley 2200 DC power supply was used to provide power to the heater. The voltage was slowly increased from 0 to 20 V in constant current (CC) mode to prevent damage to the heater. The emitted heat was observed using an IR camera, and the temperature change versus voltage was recorded.

### Sample preparation for cyclic test

For the cyclic experiment, Cu and Ni lines were printed onto a PI sheet. The printed line width was approximately 1.5 mm, and the PI sheet width was 10 mm. The specimens were cycled using an Electro Force 3200 DMA machine. The gauge length was *L*_0_ = 10 mm, and the displacements was applied from 4 mm to 8 mm based on the strain calculation for 0.9% and 1.3% cyclic strains in the printed metal. The corresponding calculated radius of curvature for 0.9% and 1.3% strain was 2.52 mm, and 1.78 mm, respectively. During the cyclic test, the electrical resistance of the patterns were measured using an Agilent 34410 A multimeter at every 1,000 cycles. 30 nm thick Ni and Cu layers were deposited by e-beam deposition on PI substrates, with ~1.5 mm width and 10 mm length. The Ni and Cu samples were cycled with the same displacement for 0.9% and 1.3% cyclic strains, and the resistance was measured at every 1,000 cycles. As e-beam deposited Ni/Cu layer is in parallel with printed metals, the resistance of the initial Ni/Cu layer was deducted from the total resistance by using parallel resistance formula similar to the resistivity calculation section.

### Supporting Information

Radius of curvature calculation, SEM images of cracks after cyclic test, tape peeling test, calibration of strain sensor and heater.

## Supplementary information

Suplementary information
