# Supplementary material for: A Hybrid Process for Printing Pure and High Conductivity Nanocrystalline Copper and Nickel on Flexible Polymeric Substrates
Source: Sci Rep. 2019 Dec 13;9:19032. doi: 10.1038/s41598-019-55640-7 (PMC6911108; doi:10.1038/s41598-019-55640-7)
Supplement: Supplementary file 1 — Suplementary information [file 41598_2019_55640_MOESM1_ESM.docx]

**Supplementary Information**

**A Hybrid Process for Printing Pure and High Conductivity Nanocrystalline Copper and Nickel on Flexible Polymeric Substrates**

*Md Emran Hossain Bhuiyan, Ali Behroozfar, Soheil Daryadel, Salvador Moreno, Reza Morsali,* and *Majid Minary-Jolandan^2^*^*^

Department of Mechanical Engineering, The University of Texas at Dallas, Richardson, TX 75080, USA

^*^E-mail: [majid.minary@utdallas.edu](mailto:majid.minary@utdallas.edu)

***Figure S1.*** *(A) and (B) Optical microscope images of microscale Cu printed on a PI substrate.*

***Figure S2.*** *(A) The cyclic flexural test of a metal pattern printed on the flexible substrate. (B) The close-up side-view of the substrate within the grips.*

**Radius of curvature and strain calculation for the cyclic test**

The radius of curvature and the developed strain in the specimen were calculated using the initial length, *L*, and the applied displacement *dL*. The shape of the curve is described by the following sinusoidal curve equation, $w=w_{o}Sin(\frac{\pi X}{L})$, where $w_{o}= \frac{2}{\pi}L \sqrt{\left( \frac{dL}{L}-\frac{\pi^{2}h^{2}}{12L^{2}} \right)}$ [[1](#_ENREF_1)] and $\frac{dL}{L} ,$*h*, *w*, and $w_{o}$ denote the applied strain, total sample thickness, deflection of the sample in the z-direction, and deflection of the sample at the center (i.e *X=* $\frac{L}{2}$) . Second derivative of the sinusoidal curve equation is the curvature of the bent, and its reciprocal is the nominal bend radius, which is given by $R$ *=* $\frac{L}{2\pi\sqrt{\frac{dL}{L}-\frac{\pi^{2}h^{2}}{12L^{2}}}}$ . Corresponding bending strain was calculated by$\epsilon= \frac{y}{R}$, where *y* is the distance between the neutral plane and the top surface of the sample. Neutral axis was calculated by considering the sample as a composite beam. Considering, the Young’s modulus of printed Ni and Cu as 200 GPa and 128 GPa respectively, and the elastic modulus of the PI sheet as 2.5 GPa, the calculated flexural strain of a 5-layer Ni sample is 0.9% using the above mentioned equations.

***Figure S3.*** *The schematic of the composite model for flexure test.*

***Figure S4.*** *SEM micrographs of the surface of printed (A) copper and (B) nickel: (I) after printing and etching, (II) after 10,000 cycles with 0.9% peak strain, and (III) after 5,000 cycles for copper and 6,000 cycles for Ni with 1.3% peak strain. C and D are the corresponding high magnification SEM images of A and B. All scale bars are 2 µm in C and D.*

To qualitatively assess the adhesion between the printed metals and the PI and PET sheets, a conventional tape-peel test was performed. The results show that overall the printed metal has a very good adhesion with the polymeric substrates. After peeling the tape from the printed lines, the printed lines remain on the surface with very little or no change, and none or only a very thin layer of the metal could be detected on the tape (Figure S5). The good bonding can be explained as follows. There are two interfaces in the samples: first, the interface between the PI substrate and the e-beam evaporated conductive Cu/Ni layer, and the second, the interface between the conductive layer and the printed Cu/Ni layers. To make the bonding between the PI substrate and the e-beam evaporated Cu/Ni layer strong, immediately prior to the Cu/Ni deposition on the PI substrate, the substrate was ultrasonically cleaned with acetone and isopropanol alcohol to make the PI surface free of contaminants. Surface roughness of the PI sheet was also helpful to achieve good adhesion. The second interface is strong since the same metal type was electrodeposited on the corresponding conductive layer, i.e. Cu on Cu and Ni on Ni. Electrodeposition involves chemical reaction, and generates metallic bonds between the printed layer and the e-beam deposited layer. Such metal-metal bond strength generally correlates with the crystallographic coherency in the interface between the film and the substrate [[2](#_ENREF_2)]. To reduce the oxidation of the initial Cu/Ni deposited films, the printing process was carried out immediately after the preparation of substrates.

***Figure S5.*** *Tape peeling test on the printed Ni and Cu on PI substrate. (A) and (B) for Ni, and (C) and (D) for Cu.*

***Figure S6.*** *The EDS spectra of printed (A) Cu and (B) Ni on PET substrate.*

**Surface roughness analysis**

***Figure S7.*** *AFM topography images of printed Ni and Cu on PI and PET substrates. Images are (5 µm × 5 µm). Before the etching step: (A) Ni printed on PI substrate, and (B) Cu printed on PI substrate. After etching: (C) Ni printed on PI substrate, (D) Cu printed on PI substrate, (E) Ni printed on PET substrate, and (F) Cu printed on PET substrate.*

***Figure S8.*** *The thickness of the metal vs. the number of printed layers: (A) Ni and (B) Cu. The surface roughness vs. the number of printed layers: (C) Ni and (D) Cu. Measured roughness at different conditions for (E) Ni and (F) Cu.*

**Calibration of the strain sensor**

***Figure S9****. (A) The experimental setup of the cantilever beam for calibration of the printed strain gauge. (B) The strain gauge is attached at the end of the beam. (C) The schematic diagram of the beam bending experiment.*

The beam bending theory was used to calibrate the strain sensor. The strain sensor was attached onto a fixed beam, which was deflected by a known distance using a micrometer at a distance *L* from the end and distance *x* from the center of the strain sensor. According to the beam theory, the bending stress is $\sigma= \frac{Mc}{I}$, where *I* is the moment of inertia of the beam about the neutral axis, *M* is the applied moment, and *c* is the distance between the cantilever surface to the neutral axis of the beam or half of the beam thickness, *t*. The bending moment at any point *x* due to the applied load, *P*, can be determined as *M = Px,* and strain can be expressed as $\varepsilon= \frac{Pxc}{EI}$. From the known maximum deflection ($\delta= PL^{3}/3EI$) of a fixed cantilever beam, the applied load $P= \delta3EI/L^{3}$ can be solved. Substituting *P* and *c = t/2* into $\varepsilon$ gives:

$$\varepsilon= \frac{3 t x}{2L^{3}}\delta$$

From the measured displacement $\delta$using micrometer, it is possible to calibrate the strain gauge by measuring the change in resistance as a result of strain at location *x* on the cantilever.

***Figure S10.*** *(A) The setup for characterization of the printed heater pattern. (B) The setup for characterization of the printed temperature sensor.*

***Figure S11.*** *Camera images show before and after etching of printed patterns. (A) and (B) for Cu, and (C) and (D) for Ni.*

**References**

1. Park, S.I., J.H. Ahn, X. Feng, S. Wang, Y. Huang, and J.A. Rogers, *Theoretical and experimental studies of bending of inorganic electronic materials on plastic substrates.* Advanced Functional Materials, 2008. **18**(18): p. 2673-2684.

2. Okamoto, N., F. Wang, and T. Watanabe, *Adhesion of electrodeposited copper, nickel and silver films on copper, nickel and silver substrates.* Materials transactions, 2004. **45**(12): p. 3330-3333.
